# Choledochal malformations in adults in the Netherlands: Results from a nationwide retrospective cohort study

**DOI:** 10.1111/liv.14568

**Published:** 2020-08-03

**Authors:** Ruben H. de Kleine, A. Marthe Schreuder, Anneke ten Hove, Jan B. F. Hulscher, Inne H. M. Borel Rinkes, Cornelis H. C. Dejong, Jeroen de Jonge, Philip de Reuver, Joris Erdmann, Geert Kazemier, the PALGA Foundation, Thomas M. van Gulik, Annette S. H. Gouw, Robert J. Porte

**Affiliations:** ^1^ Division of Hepato‐Pancreato‐Biliary Surgery and Liver Transplantation Department of Surgery University of Groningen University Medical Center Groningen Groningen the Netherlands; ^2^ Department of Surgery Cancer Center Amsterdam Amsterdam UMC University of Amsterdam Amsterdam the Netherlands; ^3^ Division of Pediatric surgery Department of Surgery University of Groningen University Medical Center Groningen Groningen the Netherlands; ^4^ Department of Surgery University Medical Center Utrecht Utrecht the Netherlands; ^5^ Department of Surgery University Medical Center Maastricht Maastricht the Netherlands; ^6^ Department of Surgery RWTH Uniklinikum Aachen Aachen Germany; ^7^ Department of Surgery Erasmus Medical Center Rotterdam the Netherlands; ^8^ Department of Surgery Radboud University Nijmegen Medical Center Nijmegen the Netherlands; ^9^ Department of Surgery University of Leiden Leiden University Medical Center Leiden the Netherlands; ^10^ Department of Surgery Cancer Center Amsterdam Amsterdam UMC Amsterdam the Netherlands; ^11^ PALGA Foundation. The nationwide network and registry of histo‐ and cytopathology in the Netherlands; ^12^ Department of Pathology University of Groningen University Medical Center Groningen Groningen the Netherlands

**Keywords:** bile duct carcinoma, choledochal cyst, choledochal malformation, surgery

## Abstract

**Background and Aims:**

Patients with a choledochal malformation, formerly described as cysts, are at increased risk of developing a cholangiocarcinoma and resection is recommended. Given the low incidence of choledochal malformation (CM) in Western countries, the incidence in these countries is unclear. Our aim was to assess the incidence of malignancy in CM patients and to assess postoperative outcome.

**Methods:**

In a nationwide, retrospective study, all adult patients who underwent surgery for CM between 1990 and 2016 were included. Patients were identified through the Dutch Pathology Registry and local patient records and were analysed to determine the incidence of malignancy, as well as postoperative mortality and morbidity.

**Results:**

A total of 123 patients with a CM were included in the study (Todani Type I, n = 71; Type II, n = 10; Type III, n = 3; Type IV, n = 27; unknown, n = 12). Median age was 40 years (range 18‐70) and 81% were female. The majority of patients (99/123) underwent extrahepatic bile duct resection, with additional liver parenchyma resections in eight patients, only exploration in two, and a local cyst resection in eight patients. Postoperative 30‐day mortality was 2% (2/123) and limited to patients who underwent liver resection. Severe morbidity occurred in 24%. In 14 of the 123 patients (11%), a malignancy was found in the resected specimen. One patient developed a periampullary malignancy 7 years later.

**Conclusions:**

In a large Western series of CM patients, 11% were found to have a malignancy. This justifies resection in these patients, despite the risk of morbidity (24%) and mortality (2%).

**Lay summary**

A Choledochal malformation is a very rare dilatation of the bile duct. To prevent the development of bile duct cancer, an operation is advised. We gathered data of 123 patients operated upon and described their diagnosis, surgery and outcome through a nationwide collaborative study.

## 1 INTRODUCTION

Choledochal malformation (CM) is a rare congenital malformation of the bile ducts and is considered a premalignant condition. CM is generally characterized by fusiform or saccular dilatation of the extrahepatic (or larger intrahepatic) bile duct(s) and an alternative (now obsolete) term for these abnormalities is choledochal cysts. The exact incidence of CM is largely unknown and is race‐dependent. Some authors report an estimated 100 to a 1000 higher incidence of CM in Asia, compared to Western countries, with a strong female preponderance.^1^, ^2^, ^3^, ^4^ In children the incidence of CM is estimated at 1 in 59 000 live births.^5^, ^6^ This is higher than previously suggested in the literature, possibly as a result of improved detection of CM with the introduction of antenatal ultrasound.^7^ The maximum diameter of the normal common bile duct has been described in the Japanese population by the JSGPM in 2016, but no norm value exists for non‐Asian populations.^8^

Because of potential malignant degeneration, standard treatment of CM consists of prophylactic resection of the affected part of the extrahepatic bile duct, including the gallbladder, followed by restoration of the biliodigestive tract via a Roux‐Y hepaticojejunostomy (HJ). With this procedure the premalignant tissue is resected to avoid the later formation of cholangiocarcinoma (CCA) or gallbladder carcinoma (GBC). It is, however, difficult to ascertain the real risk of formation of biliary malignancies because the prevalence of asymptomatic CM in the general population is unknown.

In the Western population, the incidence of CM in adults is understudied with only three studies describing cohorts larger than 36 patients and this lack of data forms the basis for our investigation.^9^, ^10^, ^11^ Extrahepatic bile duct resection (EHBDR) is associated with considerable short‐term morbidity and mortality. Long‐term complications include anastomotic strictures of the HJ, often requiring radiological and/or surgical re‐intervention. Nevertheless, detailed information on the occurrence and optimal treatment of these complications is lacking. Because of the impact of these complications, weighing of the risks and benefits of prophylactic resection poses a clinical problem for patients and surgeons.

The aim of this study was to analyse a nationwide cohort of adult patients with CM, with special interest for the occurrence of biliary malignancy associated with CM. Furthermore, we assessed the short‐term and long‐term complications of surgical therapy.

## 2 METHODS AND MATERIALS

This was a multicentre retrospective cohort study. Each of the eight academic medical centres in the Netherlands participated in the patient search. In the Netherlands, major biliary surgery, as well as surgery for congenital malformations, is performed mainly in one of these centres. The study was approved by the medical ethics committee of the University Medical Center Groningen (UMCG) and the need for patients’ informed consent was waived (reference number METC2016/341). The STROBE guideline for reporting of cohort studies was adhered to.^12^

### 2.1 Patient population and identification

A structured search of the Dutch Pathology Registry (PALGA) was performed using a selection of medical subject headings (MeSH) criteria as listed in Appendix 1. PALGA, established in 1971, is the nationwide network and registry of histo‐ and cytopathology.^13^ It prospectively collects data from all Dutch pathology laboratories and serves as a central basis for research. Conclusions of pathology excerpts of all potential cases were read and, when appropriate, excerpts were studied in more detail. If relevant to the present study, the selected pathology data were returned to each participating centre and matched with the hospital record for further analysis.

Using the same MeSH criteria, hospital‐based registries were searched for eligible patient files. Data retrieved from PALGA and from hospital‐based registries were cross‐checked to avoid duplicates and this patient identification was used for on‐site medical file evaluation.

To determine the incidence of CM, we aimed to include all consecutive adult (Age ≥ 18 years at the time of surgery) patients who had underwent a surgical resection for CM, with or without CCA, between 1990 and 2016. We excluded patients who were not operated upon and patients in whom the final diagnosis was not a CM as termed in the operation report or the postoperative discharge letter. In case of an unclear diagnosis or clear secondary, acquired dilatation of the bile ducts not related to CM, patients were also excluded. We did collect data on patients with a Type V CM but we excluded these patients from the present analysis because their disease is located deep into the liver parenchyma and its surgical treatment is strikingly different.

### 2.2 Data collection and outcome definitions

**Figure 1:**
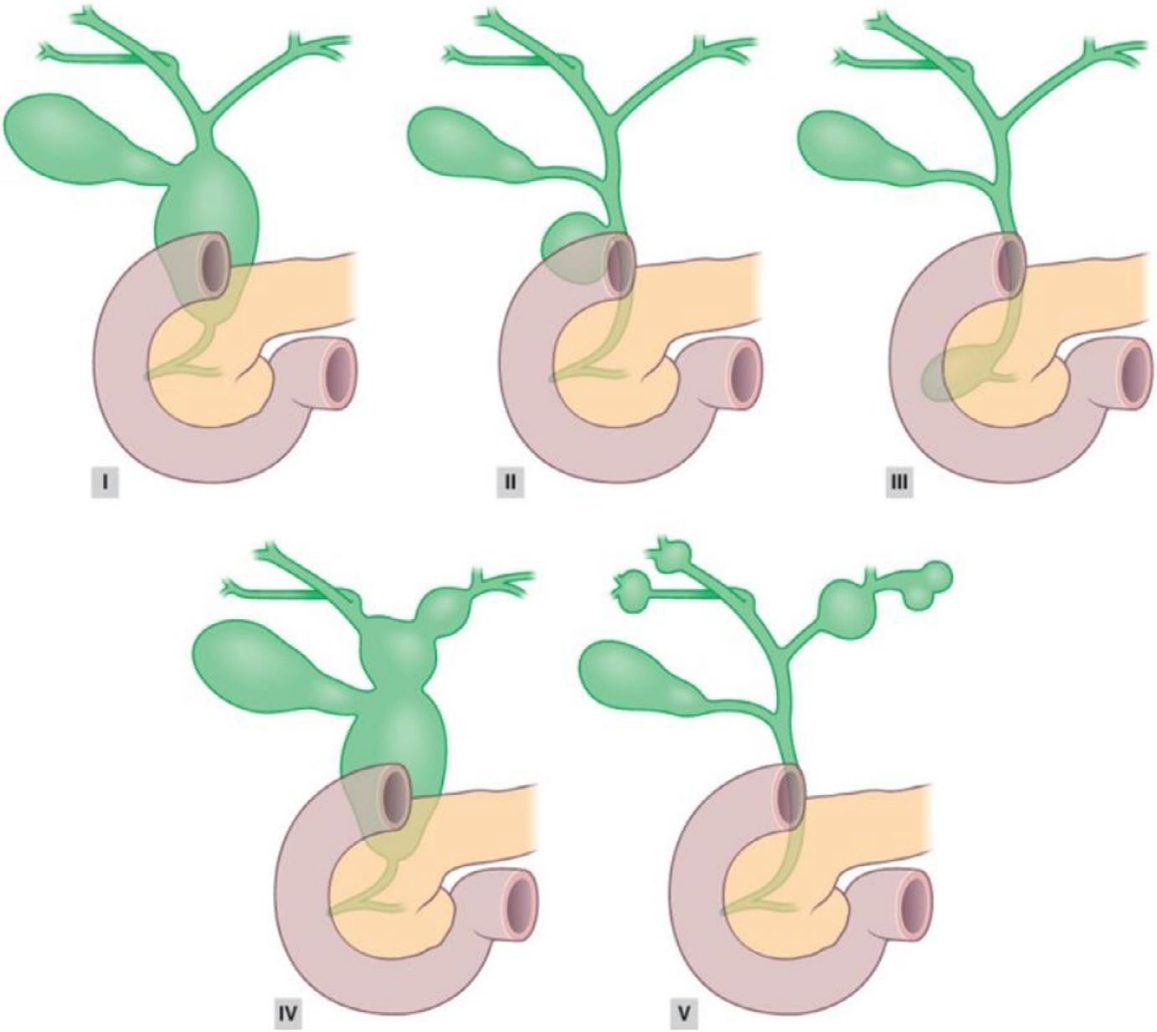
Todani classification of Choledochal malformations

**Figure 2:**
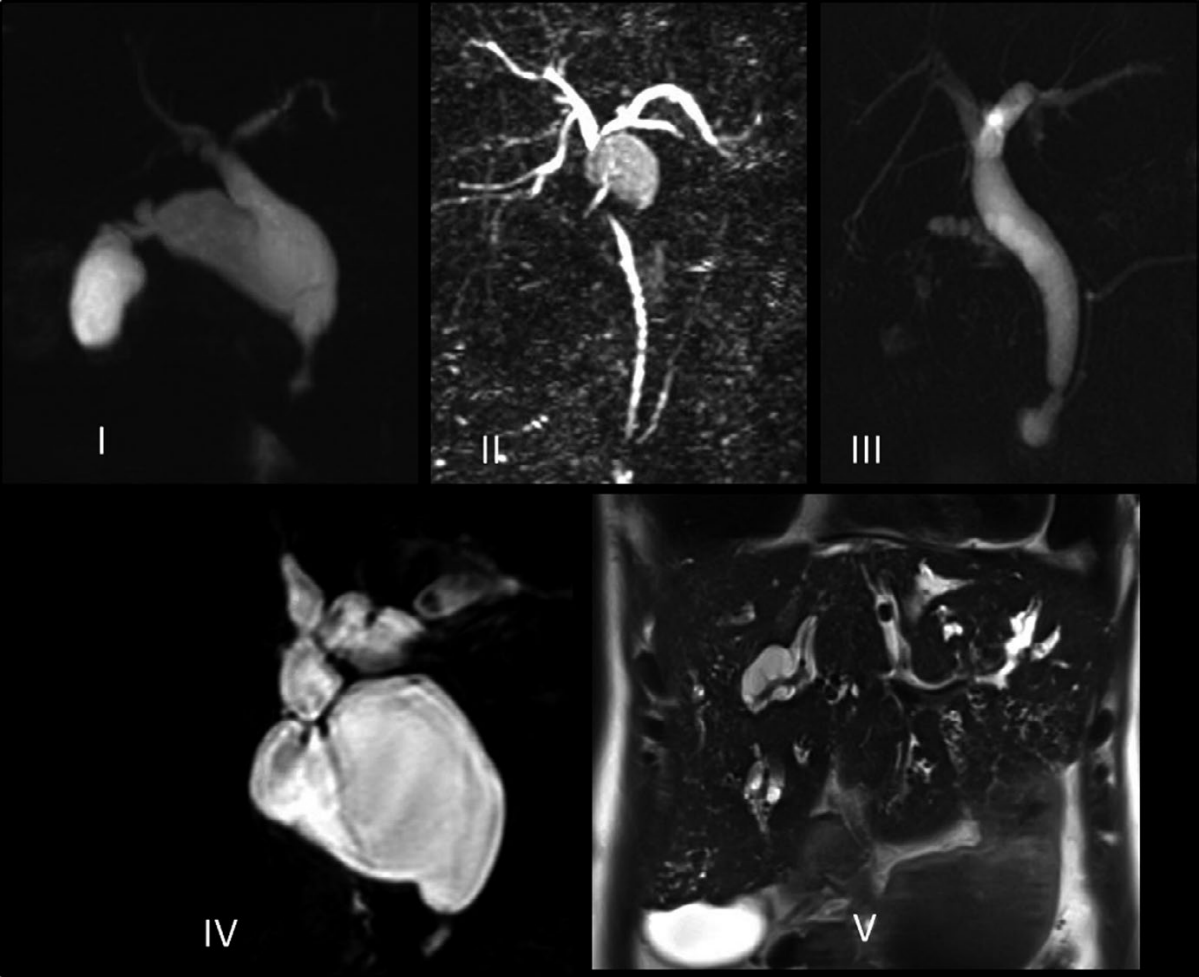
Magnetic Resonance Cholangio‐Pancreatography of matching Todani category

We extracted the data from patient charts and anonymously recorded the data using a standardised case record form (CRF, Appendix 2). This CRF was defined and maintained during the complete data acquisition. All data were collected by at least 2 researchers (either RdK, AMS and/or AtH) and disagreements between researchers were resolved by consensus. The parameters collected included patient demographics (sex, age at time of surgery and the length of time patients were followed up), presenting symptoms, type of CM according to the clinically determined Todani classification as displayed in Figures 1 and 2.^14^ The operative details, postoperative complications and the incidence of CCA or gallbladder adenocarcinoma (GBA) at the time of surgery or during follow‐up were registered.

We graded the short‐term complications (≤30 days) according to the highest Clavien‐Dindo classification.^15^ We considered short‐term complications grade 3 or higher as major complications and specified them in more detail. These included hepaticojejunostomy leakage, intra‐abdominal abscess or collection, haemorrhage (requiring an intervention) and sepsis. Postoperative mortality was defined as death by any cause within 30 days of surgery.

Long‐term complications (>30 days) included recurrent cholangitis, which we defined as jaundice with fever (>38.5°C) and abdominal pain, anastomotic stricture of the HJ, bile stones (intra‐hepatic stone formation confirmed by ultrasonography), liver abscesses (fever with bacterial infection proven via culture and infection of the liver confirmed by ultrasonography), incisional hernia requiring re‐intervention.

### 2.3 Statistics

Analytical statistics were used to compare groups within the cohort. Continuous data were presented as median with range, and categorical data as numbers with percentages. Missing data were excluded from the analysis per item. The SPSS (IBM Corporation, Armonk, NY, USA) package 26 was used for the analysis.

## 3 RESULTS

A total of 1329 excerpts of 1013 patients treated between January 1990 and December 2015 were identified through searching the PALGA database. After reading the excerpts, we considered 232 reports on 227 patients of sufficient interest to warrant detailed investigation and we subsequently matched these with local hospital records including the year 2016.

After combining the two sources of data, we identified a total of 152 adult patients with CM who had undergone surgery. From this number, we excluded 29 patients with Type V malformations. Ultimately, therefore, we analysed the data pertaining to 123 patients. This gave an overall incidence of 5.85 treated cases of CM (Type I‐V) per year, or 4.73 cases of CM Type I–IV per year. In 2000, the size of the Dutch adult population was approximately 11.9 million.^16^

### 3.1 Patient characteristics

In Table 1, we provide a list of patients’ characteristics and surgical details. Female patients formed the majority of patients (81%, 100/123). Median age at surgery was 40 years (range 18‐70). Data on presenting symptoms and associated diagnosis were missing in 13 patients. Symptoms were present in 88% of patients (98/110) and consisted mainly of abdominal pain (84%, 92/110) with several patients suffering from more than one symptom. Choledocholithiasis was present in 25% and pancreatitis in 8%. Jaundice was present in 12 upon diagnosis and resolved before surgery with endoscopic drainage in all patients. The combination of an abdominal mass, jaundice and pain was present in only one patient. No signs of liver fibrosis or splenomegaly were observed. At the moment of evaluation of the records, 15 patients had died with data missing for five patients. Ultrasound (US) was performed in 56 of 108 patients, computed tomography (CT) in 44 of 109, both US and CT in 18. Magnetic resonance imaging was performed in 69 patients and at least one imaging study was ascertained in 113, with missing data in 10. Abnormal pancreatico‐biliary maljunction was present in 22 and absent in 20, with 81 patients having missing data.

**Table 1:** Patient characteristics and operative details

	N = 123	%/range
Age (yrs, median, range)	40	18‐70
Female patients	100	81
ASA classification (n = 79^a^)		
1‐2	75	95
≥3	4	5
Presenting symptoms (n = 110^b^)		
None	12	11
Abdominal pain	92	84
Nausea	45	41
Fever	18	16
Jaundice	12	11
Abdominal mass	4	4
Cholelithiasis	27	25
Cholangitis	10	9
Pancreatitis	9	8
Malignancy	4	4
Ruptured cyst	0	0
Biliary cirrhosis	0	0
Portal hypertension	0	0
Type (Todani classification)		
I	71	58
II	10	8
III	3	2
IV	27	22
Unclear	12	10
Surgery		
Exploration, no resection	2	2
Local cyst resection (Type II malformation)	7	6
EHBDR + HJ	99	80
EHBDR + HJ +parenchymal resection:	8	7
Other	2	2
Missing	5	4
Operative time in minutes (median, IQR)	248	90‐1085

Note

Abbreviations: ASA, American Society of Anesthesiologists; EHBDR, extrahepatic bile duct resection; HJ, hepaticojejunostomy.

^a^ Data were missing on 44 patients.

^b^ Data were missing on 13 patients.

The most common of types of CM were Todani Type I with 58% (71/123) and type IV 22% (27/123). Surgical resection of the malformation was attempted in all 123 patients whereby details on the type of surgery were missing for five patients. Three patients were operated on the day of diagnosis, median time to surgery was 99 days (47‐198 days, 25‐75 percentile) Fourteen patients had a period of more than one year between the establishment of the diagnosis and surgery. Extrahepatic bile duct resection followed by HJ was performed in 80 per cent (99/123). Two explorations without resection were performed because of extensive malignancy. In seven patients, a Type II cyst was resected without the need for formal bile duct resection. EHBDR with additional parenchymal resections were performed in eight patients; these included five major liver resections, one pancreatic resection and one combination of a right‐sided hemihepatectomy with a pylorus preserving pancreatico‐duodenectomy. One patient had a conversion of a cystoduodenostomy to a formal resection of the cyst and reconstruction with HJ with a concomitant colon resection. In one instance, in 1994, a hepatico‐duodenostomy was performed because the patient lacked suitable small intestine. One cyst resection was performed and bile continuity was achieved by means of a duct‐to‐duct anastomosis, in a patient with substantial co‐morbidity. Open surgery was performed on all patients.

### 3.2 Postoperative outcome

We list postoperative outcomes that included short‐term and long‐term complications in Table 2. One half of the patients did not suffer any short‐term complications. Clavien‐Dindo Grades III and IV complications were present in one‐fifth of the patients, the majority of which consisted of sepsis and bile leakage. Two patients died postoperatively as a result of bile leakage followed by sepsis and multi‐organ failure. Neither of these patients had a malignancy in their final pathology. Relaparotomy was performed in 13 patients and nine patients required radiological or endoscopic reinterventions. The median length of stay in hospital was 10 days. There was no 90‐day mortality. No long‐term complications occurred in 64% of patients. Serious complications were present in 23 per cent of patients and half of these patients suffered recurrent cholangitis. Seventeen patients with EHBDR had an anastomotic stenosis or stricture during follow‐up. Just over half of these patients required percutaneous transhepatic drainage (eight patients, 7%) or endoscopic treatment (four patients, 3%). Surgical reinterventions after 30 days were necessary in 13% of patients (16/121) which included four revisions of the HJ.

**Table 2:** Postoperative outcomes

Short‐term outcomes	N = 123	%/range
*Missing data*	*12*	*10*
No morbidity	56	50
Mild morbidity (CD 1‐2)	28	25
Severe morbidity (CD 3)	20	18
ICU/multi‐organ failure (CD 4)	5	5
Postoperative mortality (CD 5)	2	2
Sepsis	16	14
Intra‐abdominal abscess	15	14
Hepaticojejunostomy leakage	14	13
Wound infection	9	8
Relaparotomy	9	8
Cholangitis	6	5
Haemorrhage	3	3
Length of hospital stay in days (median, range)	10	4‐69

Long‐term outcomes	N = 121	%/range
*Lost to follow‐up/ missing data*	*12*	*10*
No long‐term complication	78	72
Any long‐term complication	31	28
Anastomotic stricture/stenosis	17	16
Recurrent cholangitis	13	12
Incisional hernia	6	6
Liver abscess	1	1
(Recurrent) cholelithiasis	3	7
Secondary biliary cirrhosis	0	0
Length of follow‐up in years (median, range)	1.7	0.1 ‐ 15.7

### 3.3 Oncological outcome

Patient follow‐up was highly variable with a median length of 1.7 years (range 0.1‐15.7 years). Malignancy was proven in 14 patients (11%) by final pathology evaluation with a median age of 45.6 years (range 24.4‐68.1 years). Twelve patients were diagnosed with CCA and 2 patients with GBA. Of those, malignancy was suspected prior to surgery in 4, discovered during surgery in 6 and detected after surgery, in final pathology, in 4 patients (3%, 5% and 3% respectively). One patient who during EHBDR with HJ did not have a malignancy developed a peri‐ampullary cancer 7 years after surgery. It is unclear whether this was related to the previous CM. Cancer was only found in type I and type IV malformations. Since a more detailed evaluation of the subtypes of CM was not recorded these data were not available to us.

## 4 DISCUSSION

CM is a rare diagnosis of which the true incidence in the Western World is unknown. Based on the present nationwide cohort of 26 years, the incidence of surgically treated CM in this Western adult population of 11.9 million was 5.85 patients per year. It is highly likely that the improved detection of congenital defects with ultrasound will bring forward the detection of choledochal malformations.^6^ With improved detection and imaging, mild dilatation of the bile duct, without distal obstruction, will spark the discussion if a malformation is present. Guidelines for the Asian population by Hamada et al delineate clear age cohort maximum values for the diameter of the common bile duct but it remains to be seen if these data are useful for other populations. It is unlikely that these guidelines had an impact on clinical decisions of our cohort since they were published at the end of the treatment period.^8^

The incidence of biliary malignancy in the present cohort was 11%. Severe morbidity following resection was 22% and mortality was 2%. Leakage of the hepaticojejunostomy was detected in 14 patients (13%), higher than expected with two of these patients having additional parenchymal resections besides the EHBDR. Long‐term complications occurred in 26% of patients. This rate of complications and operative mortality seems acceptable in the light of the high risk of malignancy, especially because the majority of complications occurred in the group of patients who underwent additional parenchymal resections as a result of the suspicion or presence of cancer. Furthermore, more than half of the malignancies were not recognized preoperatively and would have been missed if patients did not undergo prophylactic surgery.

In a large cohort from the USA, the occurrence of major complications following surgery for CM was reported to be as high as 56% with a mortality rate of 7%.^11^ Another American study by Nicholl et al in 51 operated patients reported a morbidity rate of 25% with no operative mortality.^17^ Postoperative morbidity and mortality rates in surgery for cholangiocarcinoma are known to be lower in Asian countries compared to Western countries in the Western World.^18^ Accordingly, reported morbidity and mortality rates following surgery for CM are also lower in Asian series, 3%‐13% and 0%‐0.2% respectively.^19^, ^20^

The incidence of biliary malignancy found in this study is in accordance with a recent meta‐analysis reporting a prevalence of malignancy of 10.7%.^1^ Previous publications of patient series in the Western World reported varying percentages of malignancy, ranging from 3% to 19%.^9^, ^10^, ^11^, ^17^, ^21^ This may be explained by the small sample sizes of some of these studies and by the fact that these studies also included patients with Caroli's disease (Todani Type V CM). In this study magnetic resonance imaging did not display cancer in two‐thirds of those patients who, in final pathology, showed to have an adenocarcinoma. Screening for cancer in patients who opt for watchful waiting instead of prophylactic surgery remains challenging and possibly dangerous.

This patient cohort, as a result of the nationwide collaboration of academic centres, provides new insight into CM in the Western World. The completeness of the present cohort was ameliorated by identifying patients with CM by means of a systematic search of the pathology register, as well as through the local hospital registries including operative reports and discharge letters. The combination of pre‐operative imaging, operative notes (from a dedicated academic HPB surgeon) and discharge letter offers the best available retrospective tool to avoid inclusion of other diagnosis than CM. Of course, one can never be 100% certain in a retrospective study. We do, however, note limitations to the present study. First, the fact that in the past some non‐academic hospitals sporadically have performed resections for CM possibly leads to underestimation of the national incidence.^22^ Second the fact that not all patients with CM will become symptomatic and may therefore not be diagnosed at all, contributes to an underestimation of the true prevalence of CM. Third, despite the extended time span covered by our study and the multicentre collaboration, the rarity of this diagnosis kept the sample size small, prohibiting extensive statistical analysis. This also affected reliable detection of concomitant malformations like the abnormal pancreatico‐biliary maljunction or vascular malformations since no re‐evaluation of the diagnostic studies was performed. Lastly, the retrospective nature of the study, with data missing on average in 10% of patients, and the variety in length of follow‐up (on account of a lack of a structured follow‐up protocol) may have led to information bias. The findings of this study with a substantial amount of postoperative effects have led us to instate a structured yearly follow‐up with ultrasound evaluation and laboratory evaluation, even in the absence of complaints.

On account of the short duration of follow‐up, it is difficult to draw conclusions from this study on the risk of malignant transformation after resection of CM. If we include the one patient in the present study with a tumour in the periampullary region, then this risk was 0.8%. This is in line with results from several Asian patient cohorts that reported the incidence of malignant transformation after cyst resection to be 0.6%‐1.1%.^19^, ^20^, ^23^ Especially for Type IVa CM, the risk of malignant transformation is of interest, because EHBDR without liver resection, as is usually performed, would mean incomplete resection of the bile duct deformity. Future studies with longer follow‐up and intercontinental collaboration could provide more detailed insight into the factors associated with the risk of malignant transformation. Furthermore, the prognosis of patients with CM who develop a biliary malignancy should be elucidated further.

Resection of CM as a prophylactic therapy for CCA remains the pivotal recommendation to all newly detected patients with CM in view of the high risk of malignant transformation.

## CONFLICTS OF INTEREST

None reported.

## Appendix 1

**Table:**

PALGA MESH search criteria (translated into English)
Choledochalcyst
Choledochal*cyst
Ductus choledochus*cyst
Ductus choledochus*inborn error
Extrahepatic bile duct*inborn error
Choledochocele
Caroli's disease
Choledochalmalformation
Choledochal*malformation
Ductus choledochus*malformation
Cholangiocarcinoma
Bile duct carcinoma
Klatskin tumour
Choledochal*adenocarcinoma
Choledochal duct*adenocarcinoma
Bile duct*adenocarcinoma
Extrahepatic bile duct*adenocarcinoma

## References

[1] Ten Hove A , de Meijer VE , Hulscher JBF , de Kleine RHJ . Meta‐analysis of risk of developing malignancy in congenital choledochal malformation. Br J Surg. 2018;105(5):482‐490. 29480528 10.1002/bjs.10798 PMC5900735

[2] Soreide K , Korner H , Havnen J , Soreide JA . Bile duct cysts in adults. Br J Surg. 2004;91(12):1538‐1548. 15549778 10.1002/bjs.4815

[3] Singham J , Yoshida EM , Scudamore CH . Choledochal cysts: part 1 of 3: classification and pathogenesis. Can J Surg. 2009;52(5):434‐440. 19865581 PMC2769090

[4] Ragot E , Mabrut J‐Y , Ouaïssi M , et al. Pancreaticobiliary maljunctions in European patients with bile duct cysts: results of the multicenter study of the French Surgical Association (AFC). World J Surg. 2017;41(2):538‐545. 27620132 10.1007/s00268-016-3684-x

[5] van den Eijnden MHA , de Kleine RHJ , de Blaauw I , et al. Choledochal malformation in children: lessons learned from a Dutch National Study. World J Surg. 2017;41(10):2631‐2637. 28589237 10.1007/s00268-017-4064-x PMC5596029

[6] van den Eijnden MHA , de Kleine RH , de Blaauw I , et al. The timing of surgery of antenatally diagnosed choledochal malformations: a descriptive analysis of a 26‐year nationwide cohort. J Pediatr Surg. 2017;52(7):1156‐1160. 28318597 10.1016/j.jpedsurg.2017.03.003

[7] van den Eijnden MH , de Kleine RH , Verkade HJ , Wilde JC , Peeters PM , Hulscher JB . Controversies in choledochal malformations: a survey among Dutch pediatric surgeons. Eur J Pediatr Surg. 2015;25(5):441‐448. 25344941 10.1055/s-0034-1387947

[8] Hamada Y , Ando H , Kamisawa T , et al. Diagnostic criteria for congenital biliary dilation 2015. J Hepatobiliary Pancreat Sci. 2016; 23(6):342‐346. 26996969 10.1002/jhbp.346

[9] Katabi N , Pillarisetty VG , DeMatteo R , Klimstra DS . Choledochal cysts: a clinicopathologic study of 36 cases with emphasis on the morphologic and the immunohistochemical features of premalignant and malignant alterations. Hum Pathol. 2014;45(10):2107‐2114. 25123074 10.1016/j.humpath.2014.06.016

[10] Mabrut JY , Kianmanesh R , Nuzzo G , et al. Surgical management of congenital intrahepatic bile duct dilatation, Caroli's disease and syndrome: long‐term results of the French Association of Surgery Multicenter Study. Ann Surg. 2013;258(5):713‐721; discussion 21. 24121258 10.1097/SLA.0000000000000269

[11] Soares KC , Kim Y , Spolverato G , et al. Presentation and clinical outcomes of choledochal cysts in children and adults: a multi‐institutional analysis. JAMA Surg. 2015;150(6):577‐584. 25923827 10.1001/jamasurg.2015.0226

[12] Vandenbroucke JP , von Elm E , Altman DG , et al. Strengthening the reporting of observational studies in epidemiology (STROBE): explanation and elaboration. PLoS Med. 2007;4(10):e296. 17941715 10.1371/journal.pmed.0040297 PMC2020496

[13] Casparie M , Tiebosch AT , Burger G , et al. Pathology databanking and biobanking in The Netherlands, a central role for PALGA, the nationwide histopathology and cytopathology data network and archive. Cell Oncol. 2007;29(1):19‐24. 17429138 10.1155/2007/971816 PMC4618410

[14] Todani T , Watanabe Y , Narusue M , Tabuchi K , Okajima K . Congenital bile duct cysts: Classification, operative procedures, and review of thirty‐seven cases including cancer arising from choledochal cyst. Am J Surg. 1977;134(2):263‐269. 889044 10.1016/0002-9610(77)90359-2

[15] Dindo D , Demartines N , Clavien PA . Classification of surgical complications: a new proposal with evaluation in a cohort of 6336 patients and results of a survey. Ann Surg. 2004;240(2):205‐213. 15273542 10.1097/01.sla.0000133083.54934.ae PMC1360123

[16] (CBS) CBvdS . Kerncijfers bevolking. 2000 https://opendata.cbs.nl/statline

[17] Nicholl M , Pitt HA , Wolf P , et al. Choledochal cysts in western adults: complexities compared to children. J Gastrointest Surg. 2004;8(3):245‐252. 15019916 10.1016/j.gassur.2003.12.013

[18] Franken LC , Schreuder AM , Roos E , et al. Morbidity and mortality after major liver resection in patients with perihilar cholangiocarcinoma: a systematic review and meta‐analysis. Surgery. 2019;165(5):918‐928. 30871811 10.1016/j.surg.2019.01.010

[19] Lee SE , Jang JY , Lee YJ , et al. Choledochal cyst and associated malignant tumors in adults: a multicenter survey in South Korea. Arch Surg. 2011;146(10):1178‐1184. 22006877 10.1001/archsurg.2011.243

[20] Cho M‐J , Hwang S , Lee Y‐J , et al. Surgical experience of 204 cases of adult choledochal cyst disease over 14 years. World J Surg. 2011;35(5):1094‐1102. 21360306 10.1007/s00268-011-1009-7

[21] Moslim MA , Takahashi H , Seifarth FG , Walsh RM , Morris‐Stiff G . Choledochal cyst disease in a western center: a 30‐year experience. J Gastrointest Surg. 2016;20(8):1453‐1463. 27260526 10.1007/s11605-016-3181-4

[22] Bulte C , Klaase J , Russel M , Somsen J . Choledochal cyst in an adult. J Am Coll Surg. 2007;205(1):178‐179. 17617346 10.1016/j.jamcollsurg.2006.07.048

[23] Watanabe Y , Toki A , Todani T . Bile duct cancer developed after cyst excision for choledochal cyst. J Hepatobiliary Pancreat Surg. 1999;6(3):207‐212. 10526053 10.1007/s005340050108

