# Supplementary material for: Choledochal malformations in adults in the Netherlands: Results from a nationwide retrospective cohort study
Source: Liver Int. 2020 Aug 3;40(10):2469–75. doi: 10.1111/liv.14568 (PMC7540385; doi:10.1111/liv.14568)
Supplement: Supplementary file 1 [file LIV-40-2469-s001.pdf]

Case Report Form Choledochus Malformation

Registration Date *

dd-MMM-yyyy

Datum van invullen casus

Research Number *

Hospital

Sex *

☐ Female ☐ Male

Date of Birth *

dd-MMM-yyyy

Todani Conclusion

☐ Type I ☐ Type II ☐ Type III

☐ Type IV ☐ Type IVa ☐ Type IVb

☐ Type V ☐ Overige:

Pre-Operative Intrahepatic Dilatation

☐ Intrahepatic Dilatation ☐ No Intrahepatic Dilatation ☐ Missing ☐ Inapplicable

Post-Operative Intrahepatic Dilatation

☐ Intrahepatic Dilatation ☐ No Intrahepatic Dilatation ☐ Missing ☐ Inapplicable

Patient Alive

☐ Alive ☐ Deceased

Date of Death

dd-MMM-yyyy

DeathReason

☐ Complication of CM ☐ Non related disease

☐ Complication OR / pre-existent disease ☐ Malignancy related to CM

Powered by ZOHO

Adult/Child by diagnosis CM

☐ Missing ☐ Inapplicable ☐ Adult ☐ Child

Coincidence finding

☐ Missing ☐ Inapplicable ☐ Yes ☐ No

Diagnosis

Date presentation Symptoms

dd-MMM-yyyy

Date Diagnosis CM

dd-MMM-yyyy

Symptoms Diagnosis

☐ Missing ☐ Inapplicable ☐ Present ☐ Absent

Jaundice

☐ Missing ☐ Inapplicable ☐ Present ☐ Absent

Abdominal Colic Pain

☐ Missing ☐ Inapplicable ☐ Present ☐ Absent

Nausea

☐ Missing ☐ Inapplicable ☐ Present ☐ Absent

Vomiting

☐ Missing ☐ Inapplicable ☐ Present ☐ Absent

Fever

☐ Missing ☐ Inapplicable ☐ Present ☐ Absent

Biliairy Lithiasis

☐ Missing ☐ Inapplicable ☐ Present ☐ Absent

Discoloured Stool

☐ Missing ☐ Inapplicable ☐ Present ☐ Absent

Powered by ZOHO

Cholangitis

☐ Missing ☐ Inapplicable ☐ Present ☐ Absent

Pancreatitis

☐ Missing ☐ Inapplicable ☐ Present ☐ Absent

HS Megalie

☐ Missing ☐ Inapplicable ☐ Present ☐ Absent

SpontaneousRupture

☐ Missing ☐ Inapplicable ☐ Present ☐ Absent

BilairyCirrosis

☐ Missing ☐ Inapplicable ☐ Present ☐ Absent

PortalHypertension

☐ Missing ☐ Inapplicable ☐ Present ☐ Absent

Laboratory Research Diagnosis

☐ Missing ☐ Inapplicable ☐ Present ☐ Absent

Date of Lab Diagnosis

dd-MMM-yyyy

Lab Research Diagnosis

☐ ALAT

☐ ASAT

☐ GammaGT

☐ AF

☐ Tot Bilirubine

☐ Direct Bilirubine

Research Diagnosis

Research Done?

☐ Missing ☐ Inapplicable ☐ Present ☐ Absent

Powered by ZOHO

Ultrasound

- [ ] Present?
- [ ] Date
- [ ] Outcome?

ERCP

- [ ] Present?
- [ ] Date:
- [ ] Stent?
- [ ] Outcome

MRCP

- [ ] Present?
- [ ] Date:
- [ ] Outcome?

CT

- [ ] Present?
- [ ] Date:
- [ ] Outcome?

HIDA scan

- [ ] Present?
- [ ] Date:
- [ ] Outcome?

Common Channel

☐ Missing ☐ Inapplicable ☐ Present ☐ Absent

Period between Diagnosis and Operation

Presentation of symptoms

- [ ] Missing
- [ ] Inapplicable
- [ ] No Change
- [ ] New Symptoms
- [ ] Worsening of symptoms
- [ ] Less Symptoms
- [ ] No Symptoms

AbdominalPain

☐ Missing ☐ Inapplicable ☐ Present ☐ Absent

Powered by ZOHO

Icterus

☐ Missing ☐ Inapplicable ☐ Present ☐ Absent

Cholangitis

☐ Missing ☐ Inapplicable ☐ Present ☐ Absent

Pancreatitis

☐ Missing ☐ Inapplicable ☐ Present ☐ Absent

Spontaneous Rupture

☐ Missing ☐ Inapplicable ☐ Present ☐ Absent

Biliairy Cirrosis

☐ Missing ☐ Inapplicable ☐ Present ☐ Absent

Portal Hypertension

☐ Missing ☐ Inapplicable ☐ Present ☐ Absent

Biliairy Lithiasis

☐ Missing ☐ Inapplicable ☐ Present ☐ Absent

Cyst Drainage

☐ Absent ☐ -9.00 Missing ☐ -1.00 Inapplicable ☐ .00 No drainage ☐ 1.00 Drainage

Other Complications

Other Interventions

Lab results pre-Operation

Date of Last Lab pre-Operation

dd-MMM-yyyy

Powered by ZOHO

Lab Pre-Operation

- [ ] ALAT
- [ ] ASAT
- [ ] GammaGT
- [ ] AF
- [ ] Tot Bilirubine
- [ ] Direct Bilirubine

Weight (pre-OR)

OR - Operation Room

Clear Diagnosis pre-Operation

- [ ] Missing
- [ ] Inapplicable
- [ ] Unclear diagnosis
- [ ] Clear diagnosis of CM

Date of OR

dd-MMM-yyyy

Type OR

Duration OR minutes

Cyst Resection

- [ ] Missing
- [ ] Inapplicable
- [ ] Fully resected
- [ ] Absent
- [ ] Partially resected
- [ ] Not Resected
- [ ] Restant cele

Liver Resection

- [ ] Missing
- [ ] Inapplicable
- [ ] No parenchyma resection
- [ ] Parenchyma resection

Liver Aspect

- [ ] Inapplicable
- [ ] Missing
- [ ] Normal
- [ ] Not normal

Powered by ZOHO

Biopsy OR

- [ ] -9.00 Missing
- [ ] -1.00 Inapplicable
- [ ] .00 LiverBiopsy
- [ ] 1.00 Absent
- [ ] 2.00 Resected tissue
- [ ] 3.00 Resected tissue and liver biopsy
- [ ] 4.00 stones, abcecs and/or cyst fluid

PA Result Biopsy

Intra-Operative Research

- [ ] Missing
- [ ] Inapplicable
- [ ] None
- [ ] Cholangioscopy
- [ ] Cholangiogram
- [ ] Ultrasound
- [ ] Cholangiascopy + cholangiogram
- [ ] Ultrasound + Cholangiografie

BloodLoss ml

OR Complications

- [ ] -9.00 Missing
- [ ] -1.00 Inapplicable
- [ ] .00 Absent
- [ ] 1.00 present

OR Complications Description

Date Hospital Admission

dd-MMM-yyyy

Date Hospital Discharge

dd-MMM-yyyy

Profylaxe

Postoperative Treatment

Short Term COMPLICATIONS

Powered by ZOHO

Short Term Complication

- ☐ -9.00 Missing ☐ -1.00 Inapplicable ☐ .00 Absent ☐ 1.00 Present

ShortTerm Complications ClavienDindo

- ☐ -9.00 Missing
- ☐ -1.00 Inapplicable
- ☐ .00 No Complications
- ☐ 1.00 Grade 1: any deviation from normal postoperative course
- ☐ 2.00 Grade 2: Farmacological intervention
- ☐ 3.00 Grade 3: Requiring surgical/endoscopic or radiological intervention
- ☐ 4.00 Grade 4: Life threatening requiring IC/ICU
- ☐ 5.00 Grade 5: Death

Treatment ShortTerm Complication

BileLeakage ShortTerm

- ☐ -9.00 Missing ☐ -1.00 Inapplicable ☐ .00 Absent ☐ 1.00 present

Iatrogen Bowel Perforation ST

- ☐ -9.00 Missing ☐ -1.00 Inapplicable ☐ .00 Absent ☐ 1.00 present

Anostomic Leakage ShortTerm

- ☐ -9.00 Missing ☐ -1.00 Inapplicable ☐ .00 Absent ☐ 1.00 present

Wound Infection ShortTerm

- ☐ -9.00 Missing ☐ -1.00 Inapplicable ☐ .00 Absent ☐ 1.00 present

Deep Infection or Sepsis ShortTerm

- ☐ -9.00 Missing ☐ -1.00 Inapplicable ☐ .00 Absent ☐ 1.00 present

Fever Cholangitis ShortTerm

- ☐ -9.00 Missing ☐ -1.00 Inapplicable ☐ .00 Absent ☐ 1.00 present

Long Term COMPLICATIONS

Long Term YesNo

- ☐ -9.00 Missing ☐ -1.00 Inapplicable ☐ .00 Absent ☐ 1.00 present

Powered by ZOHO

LongTerm Complications CD

- [ ] -9.00 Missing
- [ ] -1.00 inapplicable
- [ ] .00 No complications
- [ ] 1.00 Grade 1: any deviation from normal postoperative course
- [ ] 2.00 Grade 2: Farmacological intervention
- [ ] 3.00 Grade 3: Requiring surgical/endoscopic or radiological intervention
- [ ] 4.00 Grade 4: Life threatening requiring IC/ICU
- [ ] 5.00 Grade 5: Death

Biliary Cirrosis

- [ ] -9.00 Missing
- [ ] -1.00 Inapplicable
- [ ] .00 Absent
- [ ] 1.00 present

Pancreatitis LongTerm

- [ ] -9.00 Missing
- [ ] -1.00 Inapplicable
- [ ] .00 Absent
- [ ] 1.00 present

Intussusception

- [ ] -9.00 Missing
- [ ] -1.00 Inapplicable
- [ ] .00 Absent
- [ ] 1.00 present

Portal Hypertension LongTerm

- [ ] -9.00 Missing
- [ ] -1.00 Inapplicable
- [ ] .00 Absent
- [ ] 1.00 present

Splenomegalie LongTerm

- [ ] -9.00 Missing
- [ ] -1.00 Inapplicable
- [ ] .00 Absent
- [ ] 1.00 present

Cholangitis LongTerm

- [ ] -9.00 Missing
- [ ] -1.00 Inapplicable
- [ ] .00 Absent
- [ ] 1.00 present

BilairyDuctStenosis

- [ ] -9.00 Missing
- [ ] -1.00 Inapplicable
- [ ] .00 Absent
- [ ] 1.00 present

Anastomic Stricture

- [ ] -9.00 Missing
- [ ] -1.00 Inapplicable
- [ ] .00 Absent
- [ ] 1.00 present

Jaundice LongTerm

- [ ] -9.00 Missing
- [ ] -1.00 Inapplicable
- [ ] .00 Absent
- [ ] 1.00 present

GallStones LongTerm

- [ ] -9.00 Missing
- [ ] -1.00 Inapplicable
- [ ] .00 Absent
- [ ] 1.00 present

Incisional Hernia LongTerm

- [ ] -9.00 Missing
- [ ] -1.00 Inapplicable
- [ ] .00 Absent
- [ ] 1.00 present

Powered by ZOHO

LiverAbcesses LongTerm

- [ ] -9.00 Missing
- [ ] -1.00 Inapplicable
- [ ] .00 Absent
- [ ] 1.00 present

Hepatic Fibrosis LongTerm

- [ ] -9.00 Missing
- [ ] -1.00 Inapplicable
- [ ] .00 Absent
- [ ] 1.00 present

Other Complications LongTerm

LongTerm Treatment

- [ ] -9.00 Missing
- [ ] -1.00 Inaplicable
- [ ] .00 absent
- [ ] 1.00 surgical
- [ ] 2.00 radiological
- [ ] 3.00 Medical

Date Long Term

dd-MMM-yyyy

Type Research LT

- [ ] -9.00 Missing
- [ ] -1.00 Inapplicable
- [ ] .00 none
- [ ] 1.00 MRCP
- [ ] 2.00 ERCP
- [ ] 3.00 X-ray
- [ ] 4.00 Ultrasound
- [ ] 5.00 PTC
- [ ] 6.00 HIDA
- [ ] 7.00 CT

Re-Treatment LT

- [ ] -9.00 Missing
- [ ] -1.00 Inapplicable
- [ ] .00 None
- [ ] 1.00 Antibiotics
- [ ] 2.00 ERCP
- [ ] 3.00 in-hospital observation
- [ ] 4.00 Operational
- [ ] 5.00 drainage
- [ ] 6.00 Medical

Date RE-Treatment

dd-MMM-yyyy

Reason Re-Treatment

Type OR Re-Treatment

Date Re-OR

dd-MMM-yyyy

Powered by ZOHO

Last LAB & Follow-Up

Date Last Laboratory

dd-MMM-yyyy

Last Lab

- [ ] ALAT
- [ ] ASAT
- [ ] GammaGT
- [ ] AF
- [ ] Tot Bilirubine
- [ ] Direct Bilirubine
- [ ] CA 19.9

Date FIRST FollowUp

dd-MMM-yyyy

Date LAST Follow-Up

dd-MMM-yyyy

Ongoing FollowUp

- [ ] -9.00 Missing
- [ ] -1.00 Inapplicable
- [ ] 1.00 Closure
- [ ] 2.00 Ongoing
- [ ] 3.00 Other Hospital

Carcinoma

Galbladder Carcinoma

- [ ] -9.00 Missing
- [ ] -1.00 Inapplicable
- [ ] .00 Absent
- [ ] 1.00 present

Info GallBladder Carcinoma

Cholangio Carcinoma

- [ ] -9.00 Missing
- [ ] -1.00 Inapplicable
- [ ] .00 Absent
- [ ] 1.00 present

Date CholangioCarcinoma

dd-MMM-yyyy

Powered by ZOHO

TNM Stage Cholangio Carcinoma

Type CholangioCarcinoma

Powered by ZOHO
